# First case of envenomation in humans caused by the banded cat-eyed snake *Leptodeira annulata* (Linnaeus, 1758) (Squamata: Dipsadidae) in Brazil

**DOI:** 10.1590/0037-8682-0555-2023

**Published:** 2024-03-25

**Authors:** Larissa Ferreira-Cunha, Mariana Fiszer, Walter Periard, Pedro Henrique Pinna

**Affiliations:** 1 Museu Nacional, Universidade Federal do Rio de Janeiro, Departamento de Vertebrados, Setor de Herpetologia. Rio de Janeiro, RJ, Brasil.; 2 Universidade de Lisboa, Faculdade de Ciências, Doutoramento em Biologia - Ecologia e Biologia da Conservação, Lisboa, Portugal.

**Keywords:** Human envenomation, Snakebite, Opisthoglyphous snake

## Abstract

A 22-year-old female researcher was bitten by a *Leptodeira annulata* on the index finger of the left hand during a contention activity. After removing the snake, a little bleeding and redness was observed in the bite region, accompanied by fang marks. Thirty minutes later, edema had progressed to the dorsum of the hand. After four hours, edema persisted, but the bitten area was slightly whitened. Treatment consisted of antibiotics and anti-inflammatory drugs. The edema resolved completely and disappeared after 48 hours. Overall, this report presents the first case of envenomation in humans caused by *Leptodeira annulata* in Brazil.

## INTRODUCTION

Ophidian accidents are relatively common worldwide, most of which involve the families Viperidae (solenoglyphous) and Elapidae (proteroglyphous)^1^. Although many opisthoglyphous snakes are disregarded as medically important, some species produce secretions through Duvernoy’s glands, which can cause physiological alterations in the victim^2^.

*Leptodeira annulata* is an opisthoglyphous Dipsadidae snake with semi-arboreal and nocturnal habits. This species occurs in Argentina, Brazil, Bolivia, Colombia, Ecuador, French Guiana, Guyana, Panama, Paraguay, Peru, and Venezuela, inhabiting low and high altitudes (from the sea level to 1,100 m elevation)^3^. Secretion of the genus *Leptodeira* causes intense proteolytic, hemorrhagic, and neurotoxic activities, damage to muscle fibers, and blockade of muscle contractions in rodents and birds^4^. Human envenomation by *Leptodeira* species includes local effects, such as intense pain, edema, hemorrhagic blistering, and erythema^2^ ^,^ ^5^. 

*Leptodeira annulata* snakebites are rare, and searches in the Cochrane Library, LILACS, SciELO, MEDLINE, PubMed, and PubMed Central databases revealed only three previous cases of human envenomation by this species, and none of them were in Brazil. Here, we report the first case of envenomation by *Leptodeira annulata* in a human in Brazil and describe its effects.

## CASE REPORT

**FIGURE 1:**
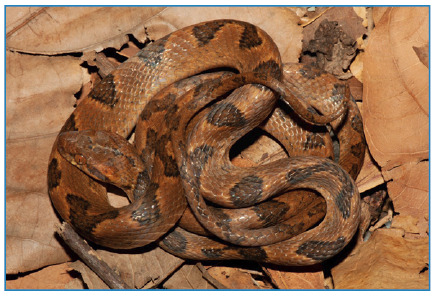
Specimen of *Leptodeira annulata* (MNRJ 27151) responsible for the accident.

On the afternoon of March 7^th^, 2019, during a contention activity, a 22-year-old female researcher was bitten on the index finger of her left hand by a female specimen of *Leptodeira annulata* (snout-vent length = 473 mm; tail length = 150.86 mm; Figure 1) from the municipality of Nova Iguaçu, Rio de Janeiro state, southeastern Brazil (22.5783° S; 43.3978° W; 50 m elevation). The specimen is now housed in the collection of reptiles at Museu Nacional, Universidade Federal do Rio de Janeiro (voucher MNRJ 27151).

**FIGURE 2:**
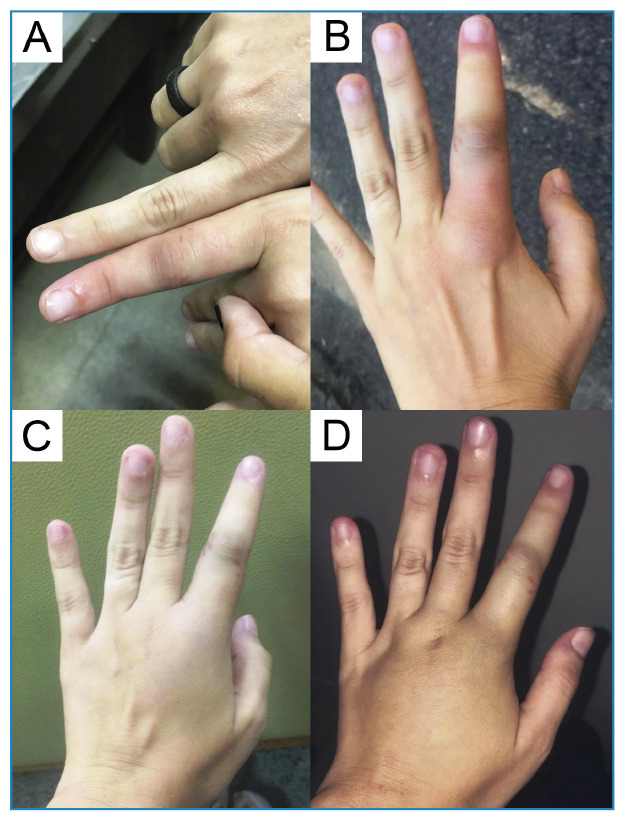
Stages of envenomation: **A.** Few minutes after the bite; **B.** Thirty minutes after the bite; **C.** One hour after the bite; **D.** Four hours after the bite.

After snake removal, the fang marks were evident, accompanied by minor local bleeding and redness. A few minutes later, the proximal interphalangeal joint was particularly affected by mild edema, redness, and moderate numbness (Figure 2A). After thirty minutes, the edema had spread out of the hand, causing ecchymosis and numbness in the metacarpophalangeal joint (Figure 2B). Approximately one hour later, the edema reached half of the dorsum of the hand (Figure 2C). The victim did not experience pain, but did have numbness and difficulty moving her finger. Four hours after the bite, the edema covered almost the entire dorsum of the hand and slightly whitened the bitten area (Figure 2D). Treatment consisted of antibiotics and anti-inflammatory agents administered for ten and three days, respectively. One day after the incident, the edema was decreased and completely disappeared after 48 hours.

## DISCUSSION

Snakebites are classified as Neglected Tropical Diseases (NTD) by the World Health Organization (WHO)^6^ owing of their impact on agricultural workers and subsistence farmers residing in rural areas. In Brazil, an average of 30,000 snakebite envenomation cases are reported annually^7^. Most of these cases (>79%) are caused by *Bothrops* species, while 8% are caused by non-venomous snakes^8^. The most commonly reported genera of non-venomous snakes associated with snakebites in Brazil are *Clelia*, *Erythrolamprus*, *Philodryas*, *Xenodon*, *Thamnodynastes, Oxyrhopus,* and *Helicops*. *Leptodeira annulata* is considered a non-aggressive snake, with only three previous cases of accidents reported in the Americas; however, this is the first case documented in Brazil. 

**FIGURE 3:**
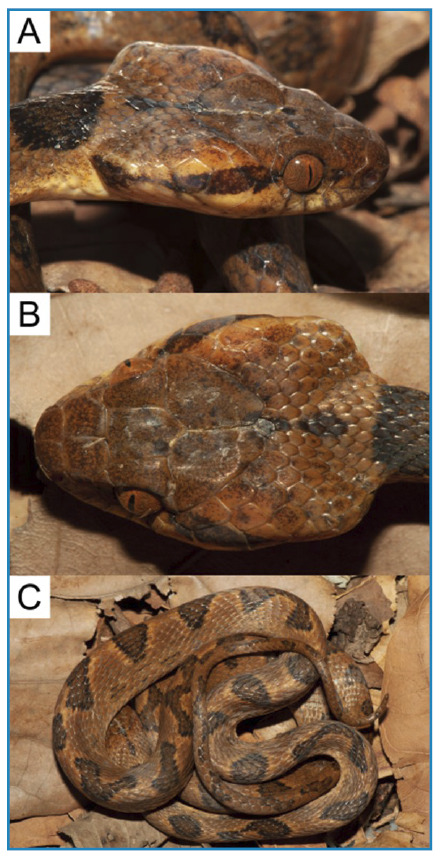
Defensive behaviors showed by the specimen of *Leptodeira annulata* (MNRJ 27151) before the bite: **A.** Head enlargement in lateral view; **B.** Head enlargement in dorsal view; **C.** Head hiding and body coiling.

Anti-predator behaviors exhibit a hierarchical pattern that follows from the consideration of relative risks, energetic demands, and intrinsic constraints, wherein aggressive defenses rarely occur without prior passive behaviors^9^. Typically, the initial response of snakes is to attempt escape, followed by the employment of passive deterrents, such as hiding the head, coiling the body, tail waving, thanatosis, or assuming a defensive ball position. If these passive deterrents fail, they may engage in aggressive defense mechanisms such as dorsoventral neck compression, head enlargement, cloacal discharge, jumping, or biting^3^ ^,^ ^9^. Additionally, many species of South American non-venomous snakes mimic species of venomous snakes, such as *Bothrops* and *Micrurus*, an indirect defense tactic^10^, as demonstrated by *Leptodeira annulata*. In the case described here, the specimen showed passive behaviors such as head enlargement (Figure 3A; 3B), followed by a combination of head hiding and body coiling (Figure 3C).

The clinical manifestations of *L. annulata* snakebites typically include mild to moderate edema in the affected area, with the possibility of spreading, erythema, and ecchymosis^3^. When envenomation occurs in the limbs, there is a possibility of partial or total loss of mobility in the fingers. Unlike the case reported in Colombia^3^, the case presented here did not involve pain or hemorrhagic blisters, suggesting that the symptoms can vary. The concentrations of enzymes in the venom of *L. annulata* vary among populations and subspecies, leading to different degrees of symptom severity^5^ ^,^ ^11^. Several factors contribute to this variation. These include the potency and volume of the toxin released during the snakebite, and the victim's individual response^1^. The morphological alterations observed probably result from the combined action of metalloproteinases (SVMPs) and phospholipase A2 (PLA2) owing to their high proteolytic and neurotoxic activity, respectively. These two enzymes are also present in the venoms of certain pit vipers, such as *Bothrops* and *Crotalus*, but PLA2 is absent in many opisthoglyphous species^5^.

The recommended initial treatment for snakebites is washing the affected area with soap and water, and taking the victim to a hospital as soon as possible. Medical treatments include anti-inflammatory drugs, antihistamines, and corticosteroids^1^. The use of prophylactic antibiotics is discouraged^12^, considering that secondary infections of reptilian origin are relatively rare (less than 1%), in contrast to the treatment administered in the present case. Similar to the current report, other ophidian accidents involving non-venomous snakes have exhibited inflammatory reactions^7^. 

The morphological mimicry and similarity in the "*Bothrops*-like" effects of the envenomation by snakes that are disregarded as medically important can result in uncertainty of medical assistance, combined with the administration of bothropic antivenom^10^. Incorrect species identification can lead to misguided treatment or absence of treatment, potentially resulting in more severe consequences or even death. Lack of knowledge regarding species identification and treatment of snakebites contributes to underestimating the true number of ophidian accidents. Environmental education and medical team training are necessary to bridge the gap in data collection on snakebite incidents and accurately determine the number of actual cases. Gathering information on the number of snakebites, envenomations, deaths, and long-term sequelae is essential to understand their impact and characteristics^7^. We emphasize the public health-relevance of publishing case reports on snakebites to enhance our knowledge of opisthoglyphous species, their symptoms, and treatments.

## References

[1] Tednes M, Slesinger TL, Tednes M, Slesinger TL (2022). Evaluation and treatment of snake envenomations.

[2] Estrella A, Navarrete L, Sánchez EE, Rodríguez-Acosta A (2011). Leptodeira bakeri (Serpentes: Colubridae): a Venomous or Non-Venomous Snake?. Russ J Herpetol.

[3] Angarita-Sierra T, Montañez-Méndez A, Toro-Sánchez T, Rodríguez-Vargas A (2020). A case of envenomation by the false fer-de-lance snake Leptodeira annulata (Linnaeus, 1758) in the department of La Guajira, Colombia. Biomedica.

[4] Lemoine K, Girón ME, Aguilar I, Navarrete LF, Rodríguez-Acosta A (2004). Proteolytic, hemorrhagic, and neurotoxic activities caused by Leptodeira annulata ashmeadii (Serpentes: Colubridae) Duvernoy's gland secretion. Wilderness Environ Med.

[5] Torres-Bonilla KA, Panunto PC, Pereira BB, Zambrano DF, Herrán-Medina J, Bernal MH (2020). Toxinological characterization of venom from Leptodeira annulata (Banded cat-eyed snake; Dipsadidae, Imantodini). Biochim.

[6] WHO. World Health Organization (2017). Report of the tenth meeting of the WHO Strategic and Technical Advisory Group for neglected tropical diseases.

[7] Kasturiratne A, Wickremasinghe AR, de Silva N, Gunawardena NK, Pathmeswaran A, Premaratna R (2008). The global burden of snakebite: a literature analysis and modelling based on regional estimates of envenoming and deaths. PLoS Med.

[8] SINAN. Sistema de Informação de Agravos de Notificação (2022). Acidente Por Animais Peçonhentos - Notificações Registradas no Sistema de Informação de Agravos de Notificação.

[9] Greene HW, Gans C, Huey RB (1988). Biology of the Reptilian.

[10] Prado-Franceschi J, Hyslop S (2002). South American colubrid envenomations. J Toxicol: Toxin Reviews.

[11] Mebs D (1970). A comparative study of enzyme activities in snake venoms. Int J Biochem.

[12] August JA, Boesen KJ, Hurst NB, Shirazi FM, Klotz SA (2018). Prophylactic antibiotics are not needed following rattlesnake bites. Am J Med.

